# Perioperative plasma glypican-3 level may enable prediction of the risk of recurrence after surgery in patients with stage I hepatocellular carcinoma

**DOI:** 10.18632/oncotarget.14271

**Published:** 2016-12-27

**Authors:** Kazuya Ofuji, Keigo Saito, Shiro Suzuki, Manami Shimomura, Hirofumi Shirakawa, Daisuke Nobuoka, Yu Sawada, Mayuko Yoshimura, Nobuhiro Tsuchiya, Mari Takahashi, Toshiaki Yoshikawa, Yoshitaka Tada, Masaru Konishi, Shinichiro Takahashi, Naoto Gotohda, Yasunari Nakamoto, Tetsuya Nakatsura

**Affiliations:** ^1^ Division of Cancer Immunotherapy, Exploratory Oncology Research and Clinical Trial Center, National Cancer Center, Kashiwa, Chiba 277-8577, Japan; ^2^ Second Department of Internal Medicine, University of Fukui, Eiheiji-cho Yoshida-gun, Fukui 910-1193, Japan; ^3^ Division of Hepatobiliary Pancreatic Surgery, National Cancer Center Hospital East, Kashiwa, Chiba 277-8577, Japan

**Keywords:** hepatocellular carcinoma, glypican-3, tumor marker, enzyme-linked immunosorbent assay, recurrence

## Abstract

Glypican-3 (GPC3) is a glycosylphosphatidylinositol-anchored cell surface protein overexpressed in hepatocellular carcinoma(HCC), and its overexpression is associated with poor prognosis. The diagnostic potential of GPC3 as a serum marker has been reported. In the present study, we evaluated the usefulness of plasma GPC3 as a predictor for recurrence after surgical resection in stage I HCC patients by newly developed an enzyme-linked immunosorbent assay (ELISA) system. Current study demonstrated that high levels of preoperative plasma GPC3 patients tended to experience postoperative recurrence. On the other hand, pre- and postoperative plasma GPC3 positivity of non-recurrence patients was very low. Moreover, even after surgery, approximately half of patients who experienced recurrence were positive for plasma GPC3. Postoperative plasma GPC3 positivity was significantly correlated with worse recurrence-free survival. Immuohistochemical analysis also showed positive rate of GPC3-expression in HCC was higher in recurrence patients than in non-recurrence patients. These results suggested that both pre- and postoperative plasma GPC3 levels may be accurate predictors for recurrence after curative resection of early-stage HCC. It should be noted that the current study only examined a small number of cases; thus, a larger sample size is necessary to validate GPC3 as a predictor for HCC recurrence.

## INTRODUCTION

Hepatocellular carcinoma (HCC) is the third leading cause of cancer-related death worldwide [1]. Patients with HCC are often diagnosed at an advanced stage, and the prognosis of these patients is generally poor. Currently, surgical resection or local radiofrequency ablation (RFA) are the standard curative treatments for early-stage HCC. However, these treatments are not highly effective, and options are limited for most patients with advanced HCC [2]. Furthermore, tumor recurrence rates remain high, even in patients who receive curative treatments, primarily because of the condition of the surrounding nontumor liver tissues, which may exhibit active hepatitis or cirrhosis [3, 4]. Thus, novel diagnostic tools to detect recurrent HCC in early stage are urgently needed.

In current therapeutic strategies, a combination of serum marker tests and imaging tests, such as ultrasonography, computed tomography (CT), and magnetic resonance imaging (MRI), is generally performed for detection of early-stage HCC. Alpha-fetoprotein (AFP) and protein induced by vitamine K absence or antagonist-II (for the latter, using [PIVKA II]) are widely used as tumor markers for detection of HCC. Many study groups have reported that preoperative levels of these tumor markers act as prognostic factors for HCC [5, 6]. However, serum AFP levels are sometimes elevated in patients without cancer who have chronic hepatitis or cirrhosis [7]. Thus, there is a need for novel tumor markers that can be used for the detection of early-stage HCC.

Glypican-3 (GPC3) is a member of the glypican family of heparan sulfate proteoglycans that are attached to the cell surface via glycosylphosphatidylinositol (GPI) anchors [8]. GPC3 is overexpressed in some types of malignant cells, including in liver cancer cells [9–13]. Additionally, GPC3 is overexpressed in 72–81% of HCC cells [14–16], but not expressed in surrounding noncancerous or cirrhotic tissues [17]. Additionally, GPC3 expression, as detected by immunohistochemical staining, has been shown to be associated with metastasis or recurrence after surgery [16, 18] and may represent a novel prognostic factor in patients with HCC after resection.

Several studies have been performed to validate the diagnostic potential of GPC3 as a serum marker in HCC [15, 19–21]. However, the diagnostic value of serum GPC3 as a predictive marker for recurrence of early-stage HCC is not well understood. Therefore, in the present study, we developed a novel enzyme-linked immunosorbent assay (ELISA) system for detecting perioperative plasma GPC3 levels and evaluated the usefulness of GPC3 as a predictor for recurrence after surgical resection, particularly in patients with stage I HCC.

## RESULTS

### Patient characteristics

Plasma GPC3 levels from 25 patients with stage I HCC who underwent surgical resection were assessed. Patient characteristics are shown in Supplementary Table 1. Of the 25 patients, 20 were male. The median age was 66.0 years (range, 41–80 years). The median alanine aminotransferase (ALT) was 47 IU/L (range, 10–131 IU/L). Twenty patients (80%) had a hepatic virus infection (two were HBV positive, and 18 were HCV positive). The median preoperative AFP and PIVKA-II were 10.9 ng/mL (range, 2–326 ng/mL) and 52 mAU/mL (range, 25–29498 mAU/mL). The mean tumor size was 39 mm (median, 30 mm; range, 14–105 mm). All patients had Child-Pugh class A disease. Hepatic fibrosis was evaluated by examination of noncancerous lesions of the resected specimen, and eleven patients were diagnosed with liver cirrhosis (LC). HCC recurrence was observed during the follow-up period after surgical resection in 14 cases (51.8%; median follow-up, 967 days; range, 99–1888 days). All of these recurrence patients had intrahepatic recurrence distant from the primary site. Solitary recurrences were occurred in 8 patients, and multiple recurrences were occurred in 6 patients. According to the Kaplan-Meier estimate, the cumulative probabilities of overall recurrence were 20.0%, 36.0%, and 58.6% at 1, 2, and 3 years, respectively. In this study, more than three years without recurrence cases after operation were defined as the non-recurrence group, and recurrence cases within follow-up period were defined as the recurrence group.

### Development of a sandwich ELISA system for detecting plasma GPC3 concentrations

A sandwich ELISA system was developed to evaluate plasma GPC3 concentrations using anti-GPC3 mAbs (10A4) as the capture antibody and biotinylated 2E11 antibodies as the detection antibody. A four-parameter logistic fit algorithm was applied to make the standard curve (R^2^ = 0.99487), which was creating using diluted recombinant GPC3. Plasma samples from 39 age-matched patients without liver disease were used as controls. In the ELISA system, the plasma GPC3 concentration (mean ± standard deviation [SD]) was 57.1 ± 50.0 ng/ml in control subjects, and a cut-off value of 132 ng/mL (mean + 1.5 SD) was established.

### Plasma GPC3 levels pre- and postoperation in patients with stage I HCC

**Figure 1:**
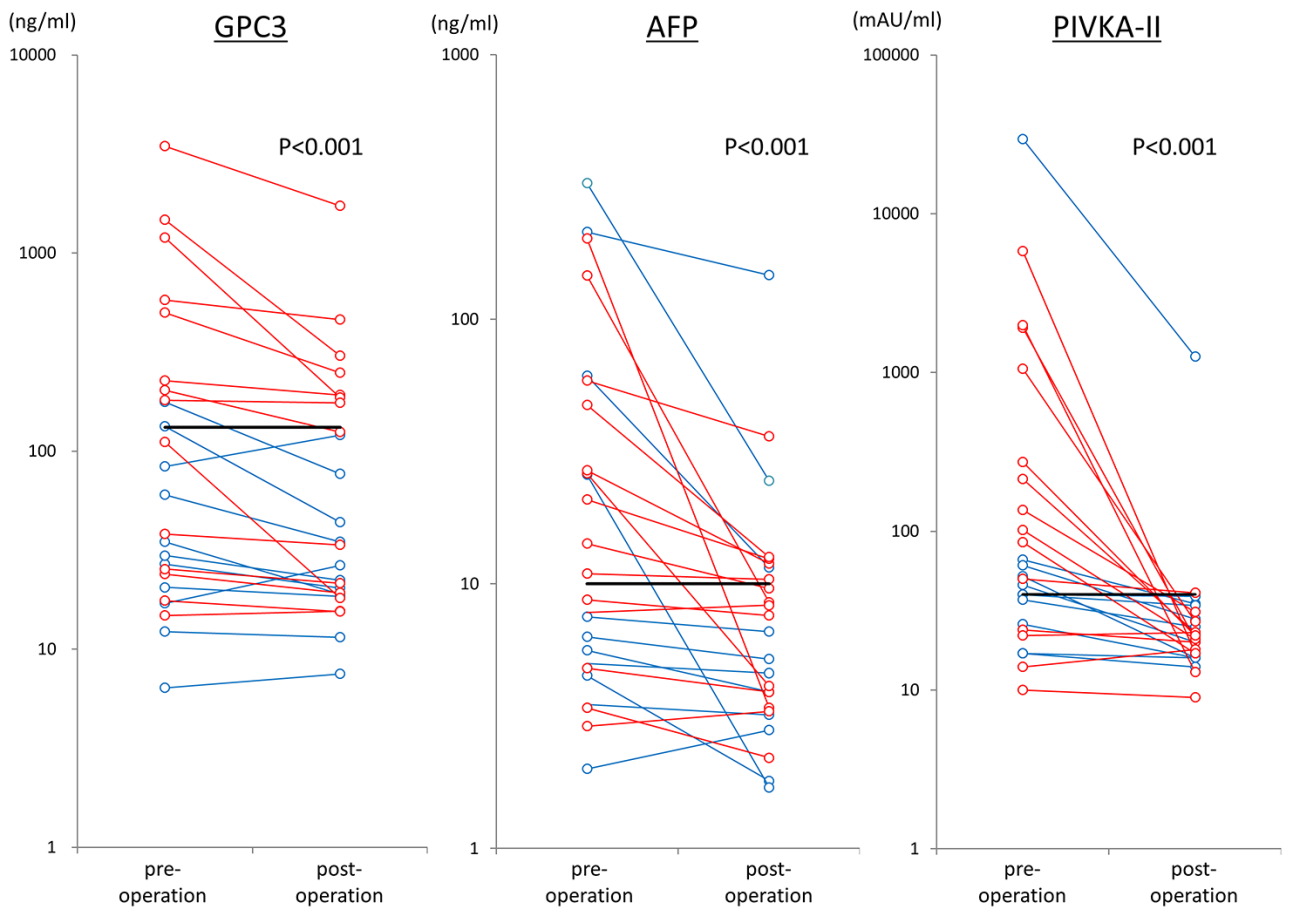
Changes in tumor markers in 25 cases before and after surgery The red lines show cases of recurrence, and the blue lines shown cases without recurrence. The black bar shows the cut off value.

Next, plasma GPC3 levels pre- and postoperation in patients with stage I HCC were measured using our newly established ELISA system. In these patients, the median preoperative plasma GPC3 level was 60.1 ng/mL (mean, 346.2 ± 747.3 ng/mL; range, 6.4–3466.8 ng/mL). Using the cut-off value of 132 ng/mL, the sensitivity and specificity of plasma GPC3 in patients with stage I HCC were 40.0% and 92.3%, respectively. Clinical changes of perioperative tumor markers are shown in Figure 1. The median postoperative plasma GPC3 level was 33.7 ng/mL (mean, 157.8 ± 346.4 ng/mL; range, 7.5–1729.3 ng/mL). Plasma GPC3 levels were significantly decreased after operation (*p* < 0.001). The postoperative plasma GPC3 level in pre-GPC3 positive LC patients(n=5) was relatively higher than that in non-LC patients(n=5) (median, 248.7 ng/mL; mean, 519.6 ± 679.5 ng/mL; range, 124.7–1729.3 ng/mL vs. median, 175.4 ng/mL; mean, 188.4± 163.4 ng/mL; range, 43.6–460.1 ng/mL), but there was no significant differences (p=0.222). Additionally, the sensitivities of preoperative AFP and PIVKA-II were 52.0% and 68.0%, respectively. Notably, the sensitivities of combination with AFP or PIVKA-II and GPC3 were increased to 72.0% or 80.0%. The sensitivity of the combination of all three markers was 88.0% (Table 1). The results from 40 cases included stage II and stage III are shown in Supplementary Table 2.

**Table 1:** Positive rates of tumor markers

		AFP	PIVKA-II	GPC3	Combination			
					AFPPIVKA-II	AFPGPC3	PIVKA-II GPC3	AFPPIVKA-IIGPC3
all cases (n=25)	pre-operation	52.0% (13/25)	68.0% (17/25)	40.0% (10/25)	88.0% (22/25)	72.0% (18/25)	80.0% (20/25)	88.0%(22/25)
	post-operation	32.0% (8/25)	8.0% (2/25)	28.0% (7/25)	32.0% (8/25)	52.0% (13/25)	36.0% (9/25)	52.0% (13/25)
non-recurrence (n=11)	pre-operation	36.3% (4/11)	63.6% (7/11)	18.2% (2/11)	81.8% (9/11)	45.5% (5/11)	63.6% (7/11)	81.8% (9/11)
	post-operation	27.3% (3/11)	9.1% (1/11)	0.0% (0/11)	27.3% (3/11)	27.3% (3/11)	9.1% (1/11)	27.2% (3/11)
recurrence (n=14)	pre-operation	64.3% (9/14)	71.4% (10/14)	57.1% (8/14)	92.9% (13/14)	92.9% (13/14)	92.9% (13/14)	92.9% (13/14)
	post-operation	35.7% (5/14)	7.1% (1/14)	50.0% (7/14)	35.7% (5/14)	71.4% (10/14)	57.1% (8/14)	71.4% (10/14)
	at recurrence	57.1% (8/14)	35.7% (5/14)	64.3% (9/14)	71.4% (10/14)	85.7% (12/14)	92.9% (13/14)	92.9% (13/14)

### Concentrations and positive rates of plasma GPC3 pre- and postoperation were high in the recurrence group

**Figure 2:**
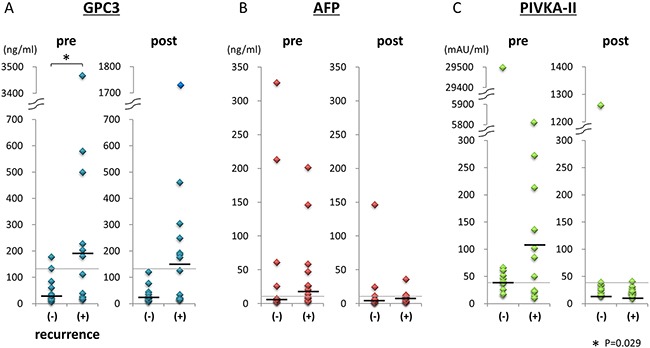
Tumor marker levels pre- and postoperation in the recurrence and nonrecurrence groups GPC3, AFP, and PIVKA-II levels pre- and postoperation in the recurrence and nonrecurrence groups are shown in **A**, **B**, and **C**, respectively. Black lines show median values of tumor markers. Gray lines showed the cut-off values.

Next, we analyzed the differences in tumor markers between the nonrecurrence (n = 11) and recurrence groups (n = 14) after surgical resection. The median preoperative GPC3 levels were significantly different between groups (*P* = 0.029; 29.7 ng/mL [mean, 54.7 ± 55.2 ng/mL; range, 6.4–176.7 ng/mL] in the nonrecurrence group; 191.7 ng/mL [mean, 575.2 ± 948.9 ng/mL; range, 14.8–3466.8 ng/mL] in the recurrence group; Figure 2A). In contrast, there were no significant differences between postoperative plasma GPC3 in the recurrence and nonrecurrence groups (mean, 253.1 ± 445.4 ng/mL; median, 150.1 ng/mL; range, 15.5–1729.3 ng/mL versus mean, 36.5 ± 33.7 ng/mL; median, 22.3 ng/mL; range, 7.5–120.3 ng/mL; *p* = 0.075). No significant correlation was observed between plasma GPC3 levels and the mode of tumor recurrence, i.e. solitary or multiple. Dot plots of pre- and postoperative AFP and PIVKA-II are shown in Figure 2B and 2C. There were no significantly differences in pre- and postoperative AFP or PIVKA-II between the nonrecurrence and recurrence groups.

Positive rates of tumor markers in the nonrecurrence and recurrence groups are shown in Table 1. In the nonrecurrence group, the GPC3 positive rates of pre- and postoperation were 18.2% and 0%, respectively. In contrast, in the recurrence group, the GPC3 positive rates of pre- and postoperation were 57.1% and 50%. Even after surgery, GPC3-positive patients were frequently identified in the recurrence group. The AFP- and PIVKA-II-positive rates postoperation were 35.7% and 7.1%, respectively, in the recurrence group. Thus, these results showed that the postoperative GPC3-positive rate was higher than the AFP- or PIVKA-II-positive rates in patients with recurrence.

### Longitudinal evaluation of plasma GPC3 levels and cases

In this study, plasma samples were obtained not only before and after operation, but also at regular intervals during follow-up in order to analyze the dynamic changes in plasma GPC3 levels. During the follow-up period, 14 patients experienced HCC recurrence after surgical resection (56%). In these patients, the median time to HCC recurrence was 575.5 days (range 99–1070 days). At the time of HCC recurrence, the median plasma GPC3 level was 169.6 ng/mL (mean, 567.5 ± 935.7 ng/mL; range, 14.6–335.1 ng/mL). The positive rates of GPC3, AFP, and PIVKA-II at the time of recurrence were 64.3%, 57.1%, and 35.7%, respectively (Table 1). The sensitivities of the combination of GPC3 and AFP or PIVKA-II were 85.7% and 92.9%, respectively.

**Figure 3:**
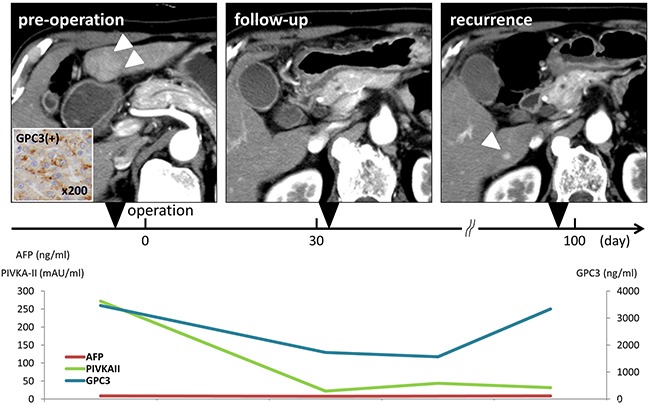
Representative patient with hepatocellular carcinoma who underwent curative operation The patient was a 72-year-old man who was HCV positive and had a 29-mm hepatocellular carcinoma in segment 3 of the liver. CT showed early enhancement of the tumor (arrowheads in the left side of the CT). Histological findings showed moderately differentiated hepatocellular carcinoma and UICC stage I. GPC3 expression in the resected specimen, as determined by immunohistochemical staining, was judged positive. Noncancerous lesions of the liver were cirrhotic. At 99 days after operation, HCC recurrence at segment 6 of the liver was diagnosed by CT (arrowhead in the right side of CT).

A representative case is shown in Figure 3. The patient was a 72-year-old man who was positive for hepatitis C virus (HCV) and had a 29-mm hepatocellular carcinoma tumor in segment 3 of the liver. He underwent operation and was diagnosed with moderately differentiated hepatocellular carcinoma according to pathological analyses. Nonvascular invasive findings were observed. Noncancerous lesions of the liver were cirrhotic. At 99 days after operation, HCC recurrence (10 mm tumor found in segment 6 of the liver) was identified by CT. Transcatheter arterial embolization (TAE) was performed for the recurrent lesion. In this patient, preoperative GPC3, AFP, and PIVKA-II were 3466 ng/mL, 8.7 ng/mL, and 272 mAU/mL, respectively. Postoperative GPC3, AFP, and PIVKA-II were 1729 ng/mL, 7.6 ng/mL, and 22 mAU/mL, respectively. At the time of recurrence, GPC3, AFP, and PIVKA-II levels were 3335 ng/mL, 9.1 ng/mL, and 32 mAU/mL, respectively. In this case, high concentrations of GPC3 were observed at all time points, including pre- and postoperation, follow-up, and recurrence. GPC3 was only positive at the time of recurrence. Consistent with this, in seven cases showing HCC recurrence, GPC3 was positive at the all time points.

### GPC3 expression by immunohistochemical staining

**Figure 4:**
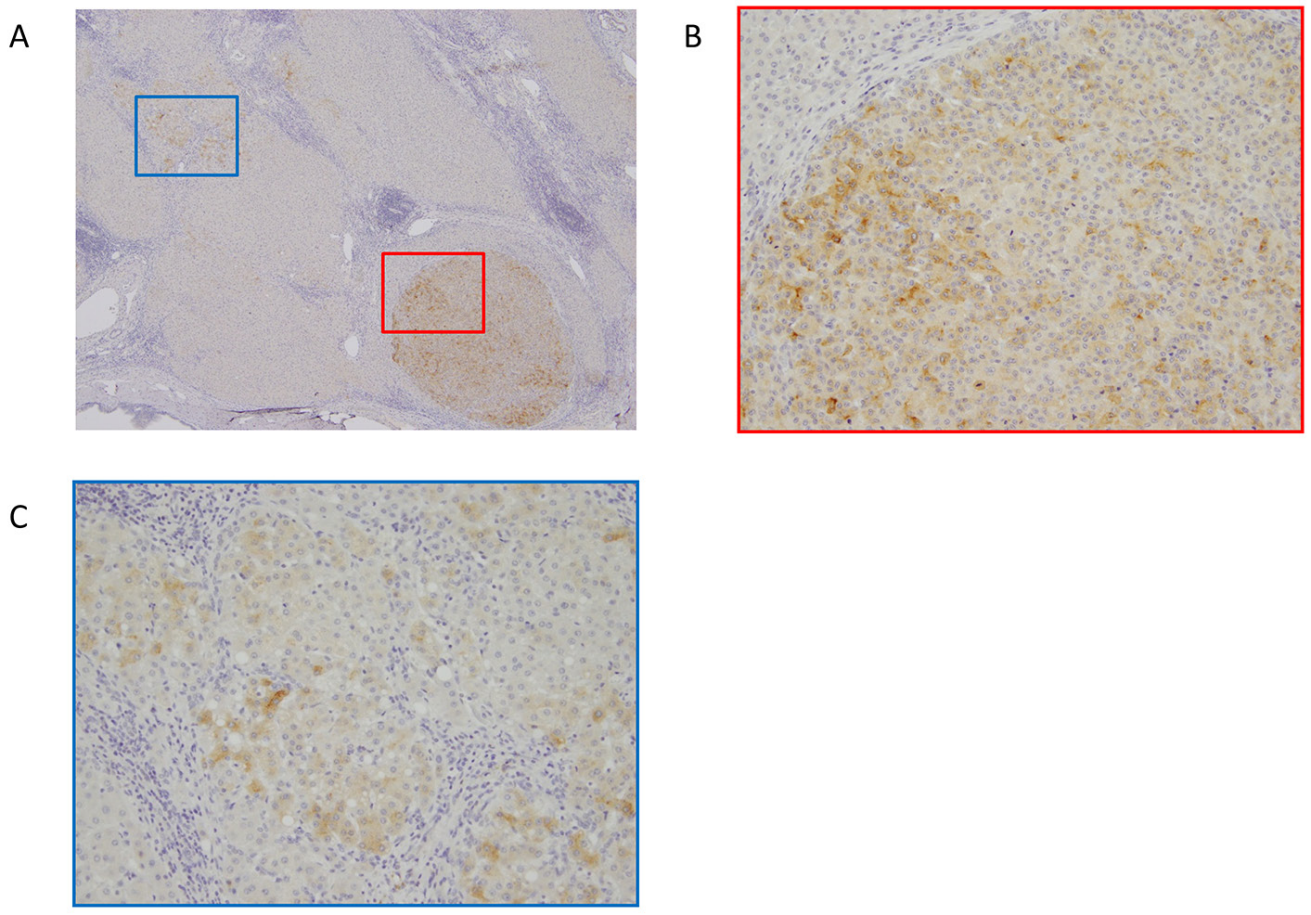
Representative immunohistochemical staining of glypican-3 (GPC3) in HCC **A**. GPC3 staining in marginal zone of HCC and surrounding non-cancerous lesion. Magnification=40X. **B**. GPC3 was expressed in HCC cells. Magnification=200X. (C) GPC3 was expressed focal and weak in non-cancerous surrounding cells. Magnification=200X.

GPC3 expression of HCC and surrounding noncancerous lesions by immunohistochemical staining was analyzed in 23 resected specimens. GPC3 expression of HCC was observed in 56.5% (13/23). 44.4% (4/9) was positive in nonrecurrence group, and 64.3%(9/14) was positive in recurrence group, respectively. In 7 speciemens, focal and weak GPC3 staining were observed in surrounding noncancerous lesions. Representative GPC3 expression in HCC and noncancerous surrounding lesion was shown in Figure 4.

### Characteristics of patients with HCC with and without recurrence

**Figure 5:**
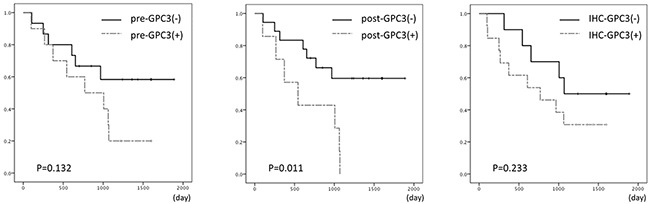
Kaplan-Meier curves for recurrence-free survival according to preoperative GPC3 (left), postoperative GPC3 (middle), and immunohistochemical GPC3 expression (right)

Next, we evaluated the prognostic factors for recurrence-free survival in patients with HCC (Table 2). Univariate analysis showed that age, gender, tumor size, hepatic virus infection, ALT, pre-AFP, pre-PIVKA-II, and GPC3 expression by immunohistochemical staining were not significant risk factors for recurrence. Hepatic fibrosis (hazard ratio [HR], 5.706; 95% confidence interval [CI], 1.700–19.153; *p* = 0.005) and postoperative plasma GPC3 positivity (HR, 3.575; 95% CI, 1.239–10.314; *p* = 0.018) were significantly correlated with the risk of recurrence. Kaplan-Meier curves for recurrence-free survival according to preoperative plasma GPC3, postoperative plasma GPC3, and immunohistochemical GPC3 expression are shown in Figure 5. There was a tendency toward a shorter recurrence-free survival in patients who were positive for GPC3 before surgery compared with that in patients who were negative for GPC3 before surgery (median RFS, 769 days versus not reached; p = 0.132), although this difference was not significant. In contrast, there were no significant differences in overall survival (*p* = 0.911). Postoperative GPC3 positivity was significantly associated with poor recurrence-free survival (median RFS, 544 days versus not reached; *p = 0.011*) and was not associated with overall survival (*p* = 0.445). Expression of GPC3 by immunohistochemical staining also tended to result in poorer recurrence-free survival (*p = 0.233*).

**Table 2:** Univariate analysis of baseline characteristics for RFS

	Non-recurrence n=11	Recurrence n=14	HR	95%CI	p value
Age (≥65/65>)	6/5	10/4	1.619	0.507-5.172	0.416
Gender (M/F)	8/3	12/2	0.633	0.141-2.833	0.550
Tumor size (≥50/50> mm)	2/9	4/10	1.478	0.462-4.732	0.510
ALT (≥40/40> IU/l)	5/6	12/2	4.248	0.947-19.063	0.059
HBV infection (+/-)	0/11	2/12	3.685	0.759-17.900	0.106
HCV infection (+/-)	7/4	11/3	1.677	0.467-6.030	0.428
Hepatic fibrosis (non-LC/LC)	10/1	4/10	5.706	1.700-19.153	**0.005**
pre-AFP (≥10/10> ng/ml)	4/7	9/5	1.998	0.666-5.992	0.217
post-AFP (≥10/10> ng/ml)	3/8	5/9	1.006	0.337-3.008	0.991
pre-PIVAKA- II (≥40/40> mAU/ml)	7/4	10/4	1.494	0.466-4.792	0.499
post-PIVAKA- II (≥40/40> mAU/ml)	1/10	1/13	0.659	0.086-5.056	0.688
pre-GPC3 (≥132/132> ng/ml)	2/9	8/6	2.152	0.745-6.126	0.157
post-GPC3 (≥132/132> ng/ml)	0/11	7/7	3.575	1.239-10.314	**0.018**
IHC GPC3 (+/-)*	4/5**	9/5	1.927	0.643-5.773	0.241

* Immunohistochemical staining of GPC3

**two cases were not available

### Relationship between GPC3 and clinicopathological parameters

The relationships among preoperative plasma GPC3 and other parameters were further analyzed in patients with stage I HCC. Positive pre-GPC3 levels (≥ 132 ng/mL) were associated with age (*p* = 0.040). Additionally, positive plasma GPC3 levels (≥ 132 ng/mL) were not associated with gender, maximum tumor size, ALT, hepatic virus infection, hepatic fibrosis, pre-AFP, pre-PIVKA-II, or GPC3 expression by immunohistochemistry (Supplementary Table 3). The relationships between positive postoperative plasma GPC3 and other parameters are shown in Supplementary Table 4. None of the examined clinicopathological parameters were associated with positive postoperative plasma GPC3.

## DISCUSSION

In the current study, we demonstrated that pre- and postoperative plasma GPC3 levels could be a useful tool for predicting recurrence after curative hepatic resection of patients with stage I HCC. We found that approximately half of patients who experienced recurrence did not show negative changes in GPC3 levels after surgery, although GPC3 levels were significantly decreased. In contrast, most patients who did not experience recurrence had negative pre- and postoperative plasma GPC3. Moreover, we found that postoperative plasma GPC3 was a significant risk factor for recurrence. A supplemental investigation of stage II and III patients showed that postoperative GPC3 positive cases were more prone to recurrence as observed with stage I patients, although the sensitivity was slightly lower. However, since only a small number of cases was examined in this study, large-scale analysis is necessary to verify these results.

GPC3 is attached to the cell surface by a glycosylphosphatidylinositol (GPI) anchor. Endogenous GPI-phospholipase D (PLD) can cleave mammalian glypicans at the level of the GPI anchor [22]. Traister et al. also demonstrated that Notum could cleave the GPI anchor and release GPC3 into the extracellular environment [23]. Thus, GPC3 may be detected in the blood stream. In addition, GPC3 can be cleaved between Arg358 and Ser359, releasing the N-terminal fragment of GPC3 into the sera [21]. Thus, several forms of secreted GPC3 could be detected in the sera. In the previous study, both full length of GPC3 and N-terminal fragment of GPC3 were detected in sera of HCC patients [15, 21]. One of the limitations of our study was that we did not determine the epitopes of the newly established anti-GPC3 mouse mAbs; thus, it is unclear how different forms of GPC3 are detected in our ELISA system was unclear. However, in our unpublished data, the same samples were measured by other ELISA system which considered to detect full-length GPC3. Similar results were obtained between two ELISA systems. Thus, we assumed that full-length GPC3 was being detected in our ELISA system.

Generally, the concentration of a tumor biomarker decreases after tumor resection or effective chemotherapy. However, in this study, we observed higher concentrations of GPC3 after operation in patients with recurrence. This tendency was not observed for AFP or PIVKA-II. One possible explanation for this high GPC3-positive rate, even in the postoperative state, may be the existence of undetectable, viable HCC lesions by imaging modalities, such as CT or MRI. These undetectable HCC cells might secrete GPC3, as discussed previously [24]. Another possible explanation is the existence of pre-neoplastic lesions in residual tissues. In the previous reports, GPC3 was not expressed in the surrounding not cancerous lesion [15, 17]. However, in the present study, GPC3 expression was observed in surrounding noncancerous lesions focal and weak. Although, characteristics of these GPC3-expressed cells were unidentified, these cells might be related to post GPC3 positive or HCC recurrence. Although a previous study showed that serum GPC3 levels were significantly higher in patients with liver cirrhosis than in healthy controls [20], we did not examine the effect of chronic liver disease on the prognostic value of GPC3 levels in HCC patients; thus, further investigation is needed to validate this measure in this subpopulation.

In the current study, we did not observe a positive correlation between preoperative GPC3 and AFP as with previous study [25]. It was reported that in 90% of AFP-negative patients *GPC3* mRNA has been shown to be overexpressed [26]. In contrast, Haruyama et al. recently reported a positive correlation between preoperative serum N-terminal GPC3 levels and AFP levels [27]. Although, the correlation between blood GPC3 levels and AFP levels is still unclear, combination strategy of AFP and GPC3 has considered to improve the sensitivity. We also found that plasma GPC3 levels were not correlated with GPC3 expression by immunohistochemical staining. The reasons for this discrepancy were unclear. One possible explanation was different anti-GPC3 mAbs were used. Another explanation was heterogeneity of GPC3 expression in HCC cells [28]. Additionally, it is possible that undetectable GPC3-secreting HCC cells may exist. Because patients who were found to be positive for GPC3 after resection also exhibited frequent recurrence, this discrepancy may be reasonable.

In conclusion, we have developed a new sandwich ELISA system to detect plasma GPC3 concentrations and showed that pre- and postoperative GPC3 levels were important risk factors for recurrence after curative resection. High levels of postoperative GPC3 indicated the existence of residual tumors that were not detectable by imaging, or existence of GPC3-expressed pre-neoplastic cells. Adjuvant treatment and close follow-up were needed in these patients. Nevertheless, the analysis of perioperative GPC3 has potential to result in identification of improved approaches for HCC diagnosis and treatment.

## MATERIALS AND METHODS

### Plasma samples

Plasma samples were collected from 25 patients with stage I HCC who underwent surgical resection at the National Cancer Center East, Japan between 2008 and 2010. All patients were pathologically diagnosed as having stage I HCC after surgery. Pathological stage was determined according to the tumor-node-metastasis (TNM) classification of the UICC [29]. Plasma samples from patients with stage II (n = 9) or stage III (n = 6) HCC were also collected, although our primary analysis focused on stage I disease. Plasma samples were collected before and after operation. The mean number of days to collect postoperative plasma samples was 25 days after surgical resection. Postoperative plasma samples were also obtained after long-term follow-up. Plasma samples from 39 age-matched patients without liver disease were also collected as controls. All samples were frozen and stored at -80°C until measurement. Written informed consent was obtained from all patients.

### Measurement of serum AFP and PIVKA-II concentrations

Serum AFP and PIVKA-II concentrations in patients with HCC were measured at the time of plasma collection for detection of GPC3 using a commercially available electrochemiluminescence immunoassay kit (Roche Co., Tokyo, Japan) and a chemiluminescent enzyme immunoassay kit (Eisai Co., Tokyo, Japan), respectively. In this study, the cut-off values for AFP and PIVKA-II were set as 10 ng/mL and 40 mAU/mL, respectively.

### Generation of anti-GPC3 monoclonal antibodies (mAbs)

Recombinant GPC3 (R&D Systems, Minneapolis, MN, USA) was used an antigen. All animal protocols were approved by the Animal Care and Use Committee of the National Cancer Center, Japan, and the experiments were performed in accordance with the institutional guidelines. Eight-week-old BALB/c mice (Charles River, Japan) were immunized with an emulsion of recombinant GPC3 (10 μg/50 μL) and 50 μL Titer Max Gold (Titer-Max USA Inc., Norcross, GA, USA), followed by repeated immunization with 10 µg of recombinant GPC3 in phosphate-buffered saline (PBS). Spleen cells were isolated and fused with mouse myeloma cells (SP2/O-Ag14; RIKEN BRC, Ibaraki, Japan) using PEG1500 (Roche Diagnostics GmbH, Mannheim, Germany). Hybridomas producing GPC3 mAbs were selected by ELISA, and cloning was performed by the limited dilution method. Twelve anti-GPC3 mAbs were generated; of these, we used two anti-GPC3 mAbs (10A4 and 2E11). Specifically, 10A4 was used as a capture antibody, and 2E11, which was biotinylated using EZ-Link Sulfo-NHS-LC-Biotin reagent (Thermo Fisher Scientific, San Jose, CA, USA), was used as a detection antibody.

### Sandwich ELISA system for detection of plasma GPC3 levels

To generate a sandwich ELISA system to measure plasma GPC3 levels, a 96-well Maxisorp ELISA plate (Nunc, Roskilde, Denmark) was coated with 0.25 μg of anti-GPC3 mAbs (10A4) in 50 μL of PBS per well. After 2 h of incubation at room temperature, 200 μL of 2% BLOCK ACE (DS Pharma Biomedical, Tokyo, Japan) in PBS was added to block the nonspecific binding at room temperature for 1 h. After washing with 0.05%Tween-20 in PBS three times, 50 μL of plasma, diluted 1:10 in PBS, or recombinant GPC3 as a standard was added to each well, and the plates were incubated at 4°C for 12 h. After washing two times, 50 μL of biotinylated-anti-GPC3 mAb (2E11) solution (5 μg/mL) was added at room temperature for 2 h. Plates were then washed four times, and 50 μL of horseradish peroxidase-labeled streptavidin (1 mg/mL; Thermo Fisher Scientific) diluted 1:16000 in 0.4% BLOCK ACE was added to each well. Plates were then incubated at room temperature for 30 min and washed. Next, 50 μL of 1-step Ultra TMB-ELISA (Thermo Fisher Scientific) as the substrate and 0.18 M H_2_SO_4_ were added. The absorbance at 450 and 540 nm was measured using a Bench Mark-Plus microplate spectrophotometer (Bio-Rad, Hercules, CA, USA). Recombinant GPC3 was used as a standard. The ELISA was suitable for quantification of plasma GPC3 with a range from 0 to 100 ng/mL; in this study, a range from 0 to 1000 ng/mL was measurable because we used plasma samples diluted 1/10.

### Immunohistochemical analysis

Resected specimens were stained with hematoxylin and eosin or monoclonal antibodies against GPC3 (clone 1G12; dilution 1:300; BioMosaics Inc., Burlington, VT, USA), according to the manufacturers’ directions. In this study, of 25 resected specimens, 23 specimens were analyzed.

### Statistical analysis

Quantitative pre- and postoperative values of tumor markers were compared using the Wilcoxon signed-rank test. The differences between the two groups were examined for statistical significance using Mann-Whitney U tests or Fisher's exact tests. The survival curves for overall survival (OS) and recurrence-free survival (RFS) were analyzed using the Kaplan-Meier method, and the differences were evaluated using log-rank tests. Factors associated with HCC recurrence risk were determined by the Cox proportional hazard model. All statistical analyses were performed using SPSS version 20. Differences or associations with *P* values of less than 0.05 were considered significant.

## SUPPLEMENTARY MATERIALS TABLES



## Abbreviations

GPC3: glypican-3
HCC: hepatocellular carcinoma
ELISA: enzyme-linked immunosorbent assay
RFA: radiofrequency ablation
AFP: alpha-fetoprotein
PIVKA II: protein induced by vitamin K absence or antagonist-II
TAE: transcatheter arterial embolization
