# Supplementary material for: Perioperative plasma glypican-3 level may enable prediction of the risk of recurrence after surgery in patients with stage I hepatocellular carcinoma
Source: Oncotarget. 2016 Dec 27;8(23):37835–44. doi: 10.18632/oncotarget.14271 (PMC5514954; doi:10.18632/oncotarget.14271)
Supplement: Supplementary file 1 [file oncotarget-08-37835-s001.pdf]

Perioperative plasma glypican-3 level may enable prediction of the risk of recurrence after surgery in patients with stage I hepatocellular carcinoma

SUPPLEMENTARY TABLES

Supplementary Table S1: Patient characteristics

Age (median, range)	66 (41-80)
Gender (M/F)	20/5
ALT (IU/l) (median, range)	47 (10-131)
Hepatic virus infection (NBNC/HBV/HCV)	5/2/18
AFP (ng/ml) (median, range)	10.9 (2-326)
PIVKA- II (mAU/ml) (median, range)	52 (25-29498)
GPC3 (ng/ml) (median, range)	60 (6-3466)
Tumor size (mm) (mean, range)	39 (14-105)
Stage (I/II,III,IV)*	25/0
Child-Pugh (A/B,C)	25/0
Hepatic fibrosis (non-LC/LC)	14/11

*Stage: TNM classification for HCC (Union for International Cancer Control: UICC)

Supplementary Table S2: Positive rates of tumor markers in stage I~III HCC patients

		AFP	PIVKA-II	GPC3	Combination			
					AFP PIVKA-II	AFP GPC3	PIVKA-II GPC3	AFP PIVKA-II GPC3
all cases (n=40)	pre-operation	57.5% (23/40)	72.5% (29/40)	32.5% (13/40)	90.0% (36/40)	70.0% (28/40)	85.0% (34/40)	90.0% (36/40)
	post-operation	35.7% (14/40)	15.0% (6/40)	25.0% (10/40)	35.7% (14/40)	47.5% (19/40)	47.5% (19/40)	47.5% (19/40)
non-recurrence (n=14)	pre-operation	35.7% (5/14)	71.4% (10/14)	14.3% (2/14)	85.7% (12/14)	42.9% (6/14)	71.4% (10/14)	85.7% (12/14)
	post-operation	21.4% (3/14)	7.1% (1/14)	0.0% (0/14)	21.4% (3/14)	21.4% (3/14)	7.1% (1/14)	21.4% (3/14)
recurrence (n=26)	pre-operation	69.2% (18/26)	73.1% (19/26)	42.3% (11/26)	92.3% (24/26)	84.6% (22/26)	92.3% (24/26)	92.3% (24/26)
	post-operation	42.3% (11/26)	26.4% (5/26)	38.5% (10/26)	42.3% (11/26)	61.5% (16/26)	53.8% (14/26)	61.5% (16/26)
	at recurrence	56.0% (14/25)	44.0% (11/25)	46.2% (12/26)	72.0% (18/25)	69.2% (18/26)	80.8% (21/26)	80.8% (21/26)

Supplementary Table S3: Relationship of positive preoperative GPC3 and clinicopathological parameters

	Cases	pre-GPC3 positive case	pre-GPC3 negative case	P value
Age				P<0.05
≥65	16	9	7	
65>	9	1	8	
Gender (M/F)				n.s.
Male	20	8	12	
Female	5	2	3	
Tumor size				n.s.
≥50 mm	6	2	4	
50> mm	19	8	11	
ALT				n.s.
≥40IU/l	17	9	8	
40> IU/l	8	1	7	
HBV infection				n.s.
positive	2	0	2	
negative	23	10	13	
HCV infection				n.s.
positive	18	8	10	
negative	7	2	5	
Hepatic fibrosis				n.s.
non-LC	14	5	9	
LC	11	5	6	
pre-AFP				n.s.
≥10 ng/ml	13	5	8	
10> ng/ml	12	5	7	
pre-PIVAKA- II				n.s.
≥40 mAU/ml	16	7	9	
40> mAU/ml	9	3	6	
IHC GPC3				n.s.
positive	13	6	7	
negative	10	4	6	

Supplementary Table S4: Relationship of positive postoperative GPC3 and clinicopathological parameters

	Cases	post-GPC3 positive case	post-GPC3 negative case	P value
Age				n.s.
≥65	16	6	10	
65>	9	1	8	
Gender (M/F)				n.s.
Male	20	7	13	
Female	5	0	5	
Tumor size				n.s.
≥50 mm	6	2	4	
50> mm	19	5	14	
ALT				n.s.
≥40IU/l	17	7	10	
40> IU/l	8	0	8	
HBV infection				n.s.
positive	2	0	2	
negative	23	7	16	
HCV infection				n.s.
positive	18	6	12	
negative	7	1	6	
Hepatic fibrosis				n.s.
non-LC	14	3	11	
LC	11	4	7	
pre-AFP				n.s.
≥10 ng/ml	13	3	10	
10> ng/ml	12	4	8	
pre-PIVAKA- II				n.s.
≥40 mAU/ml	16	5	11	
40> mAU/ml	9	2	7	
IHC GPC3				n.s.
positive	13	4	9	
negative	10	3	7	
